# The Blood-Pressure during Pregnancy and the Puerperium

**Published:** 1910-09-17

**Authors:** 


### GYNÆCOLOGY.

### THE BLOOD-PRESSURE DURING PREGNANCY AND THE PUERPERIUM.

From the point of view of a case suffering from cardiac affection whether of valvular or of myocardial origin, it is of interest to know what additional work is thrown upon the heart by alterations in the blood-pressure during pregnancy and puerperium.

When the Riva-Rocci method of determining the blood-pressure is employed it has been found that under normal conditions the blood-pressure during pregnancy may vary within comparatively wide limits, though it should never be higher than 150 millimetres of mercury, or lower than 100. Upon the whole there is no difference between primiparæ and multiparæ in this respect, nor does age make any material difference; generally speaking it may be said that during the earliest months of pregnancy the pressure is about normal, gradually rising during the last eight weeks, and reaching the maximum at the beginning of the week before delivery, after which there is a slight gradual decline.

Soon after labour sets in there is a rise, increasing with each labour pain, but falling slightly in the intervals between the pains. The increase reaches its highest point just before the birth of the child, and soon after delivery there is a rapid drop, unless there has to be any intrauterine manipulation, in which case there will probably be a return to a higher level of blood-pressure. There is no doubt that the more severe the pain, and the greater the struggling and the arm movements, the greater is the blood-pressure likely to be; but even under an anæsthetic the curve of blood-pressure is very similar to that which follows when no anæsthetic is given, so that the rise is an essential part of labour. After delivery the falling pressure continues for some hours, reaching its lowest point about the eighth hour, and thereafter remaining low, with a tendency to rise gradually again, until it returns to normal in from ten to fifteen days.

The only cases of extreme hypertension are those of eclampsia, the high blood-pressure sometimes anteceding the convulsions and thus affording an early indication of the danger of the case. It is noteworthy that the blood-pressure is by no means necessarily abnormal whenever albumin is present in the urine, and as far as can be judged from most of the cases hitherto recorded determinations of the blood-pressure in cases of pregnancy with albuminuria, especially in those that are near full term, enable one to distinguish those which are not in danger from those which are, according as the blood-pressure is below 150 millimetres of mercury upon the one hand, and above 150 millimetres of mercury on the other. The occurrence of pyrexia during the puerperium does not materially affect the pressure, though one might have expected perhaps that the readings would have fallen as the result of the toxæmia. As a matter of fact marked hypotension, that is to say a falling of the blood-pressure below 100 millimetres of mercury, is to be noticed chiefly as the result of hæmorrhage.

